# Topic Analysis and Mapping of Tuberculosis Research Using Text Mining and Co-Word Analysis

**DOI:** 10.1155/2022/8039046

**Published:** 2022-11-08

**Authors:** Meisam Dastani, Alireza Mohammadzadeh, Jalal Mardaneh, Reza Ahmadi

**Affiliations:** ^1^Infectious Diseases Research Center, Gonabad University of Medical Sciences, Gonabad, Iran; ^2^Department of Microbiology, School of Medicine, Infectious Diseases Research Center, Gonabad University of Medical Sciences, Gonabad, Iran; ^3^School of Medicine, Infectious Diseases Research Center, Gonabad University of Medical Sciences, Gonabad, Iran

## Abstract

Tuberculosis is still one of the most severe progressive diseases; it severely limits the social and economic development of many countries. In the present study, the topic trend of scientific publications on tuberculosis has been examined using text mining techniques and co-word analysis with an analytical approach. The statistical population of the study is all global publications related to tuberculosis. In order to extract the data, the Scopus citation database was used for the period 1900 to 2022. The main keywords for the search strategy were chosen through consultation with thematic specialists and using MESH. Python programming language and VOSviewer software were applied to analyze data. The results showed four main topics as follows: “Clinical symptoms” (41.8%), “Diagnosis and treatment” (28.1%), “Bacterial structure, pathogenicity and genetics” (22.3%), and “Prevention” (7.84%). The results of this study can be helpful in the decision of this organization and knowledge of the process of studies on tuberculosis and investment and development of programs and guidelines against this disease.

## 1. Introduction

Tuberculosis is a chronic infectious disease that seriously affects human health [1]. According to the World Health Organization (WHO), in 2017 about 10 million people were infected with *Mycobacterium tuberculosis*, and approximately 1.3 million died from tuberculosis. With the prevalence of HIV-related tuberculosis and drug-resistant tuberculosis in recent years, this disease remains one of the most severe progressive epidemics [2].

According to the Sustainable Development Goals of the United Nations, the tuberculosis epidemic must end before 2031 [3]. Modeling studies indicate that severe reductions in the incidence of tuberculosis require new diagnostic tests, drugs, and vaccines [4]. Accordingly, research and innovation are among the main factors in the strategy of WHO to end tuberculosis epidemics. Therefore, WHO has developed a global action framework for tuberculosis research to strengthen the performance of high-quality investigations in this field from 2016 to 2025 at global and national levels [5].

Many studies have been conducted on tuberculosis worldwide, the results of which have been aimed at increasing current knowledge about the disease, updating information, and identifying present problems and challenges [6]. Since publications on this subject have been on the rise in recent years [5, 7], it is important to identify the topic trend of this research to determine the status of scientific publications and research gaps in this area. Accordingly, it is necessary to employ methods and techniques to review the scientific publications on tuberculosis to provide different types of analysis of the publications. The text mining method involves a set of procedures such as natural language processing, information retrieval and extraction, statistics, and machine learning. Clustering, categorizing, summarizing, and discovering relationships between concepts are some of the applications of text mining [8].

Topic modeling is a text mining technique [9] that examines documents to identify their themes or topics. The topic modeling results can be applied to analyze how topics relate to each other and how they evolve over time [10]. Since a topic is defined as a probability distribution over words, topic models are based on the idea that documents consist of a set of topics [11].

Co-word analysis is another frequently applied and common method for analyzing the knowledge structure in different areas that investigate the relationship between the words used in different parts of documents [title, abstract, keywords, etc.]. Co-word analysis is a type of cooccurrence analysis and is among the important bibliometric methods applied to map the relationship between concepts, ideas, and problems in the basic and social sciences [12].

By applying this type of analysis, the main topics of the studied field, semantic structure, and evolution of those works can be determined over time. In the co-word analysis, the assumption is that the most frequent words have a higher impact on a subject area than the less frequently used ones. Moreover, the co-word analysis makes it possible for researchers to identify emerging topic clusters as well as developed ones to predict the direction of future research [13].

Previous studies have identified the trend of scientific publications on tuberculosis and the contribution of countries as well as various organizations in this area [5, 7, 14–16]. Fonseca et al. analyzed the tuberculosis publications of the Fiocruz Institute of Brazil using social media analysis techniques. They identified influential and key investigations of the institute and central institutions in scientific cooperation [17]. Igwaran and Edoamodu identified the subject areas of tuberculosis publications in Africa and demonstrated the key authors, countries, institutions, and scientific collaborations in this field [6].

According to the literature, there was no study on identifying topic clusters of global scientific publications on tuberculosis. Therefore, the present study identifies the global scientific publications on the subject area of tuberculosis in the Scopus citation database and examines the subject area of this disease using topic modeling and co-word analysis.

## 2. Materials and Methods

The statistical population was all original articles on tuberculosis in the Scopus citation database. Due to its comprehensiveness for different fields of science and indexing of numerous articles, this database is appropriate for scientometric studies [18–20]. In order to determine the main keywords for searching in the Scopus database, the Medical Subject Headings (MESH) was used after consultation with experts in the field of microbiology. In the next step, using the advanced search section of the Scopus database, the publications on tuberculosis were extracted on Jan. 4, 2022, using the combination of keywords “Tuberculoses,” “Kochs Disease,” “Koch's Disease,” “Koch Disease,” “Mycobacterium tuberculosis Infection,” “Mycobacterium tuberculosis Infections,” and “Tuberculosis.” The range of 1900 to 2021 was the period considered in the search strategy, and the data were extracted in the form of a CSV file. In this research, the format of documents was limited to original articles, which were extracted from original research methods (experiments, observations, surveys, interviews, and questionnaires), and the results of these studies were indicated [21]. For this reason, only this type of article is considered in the present investigation.

After data extraction, text mining techniques and a topic modeling algorithm were applied to identify the issues of published articles on tuberculosis. The following steps have been performed.

1. For the text mining process, the titles, abstracts, and keywords of each retrieved publication were merged. Afterward, preprocessing and data cleansing operations were applied to the studied data to increase data quality, the validity of patterns, and extracted relationships [22]
2. Then, the topic modeling algorithm named Latent Dirichlet Allocation (LDA) was used. The LDA is one of the most important approaches for topic modeling [23]. It is among the best and extensively used algorithms and is significantly effective in identifying related semantic topics in scientific texts [23]

Since the LDA cannot determine the number of appropriate topics, the CV coherence measure was used, which is essentially an index that measures the cooccurrence of the words extracted by the topic model. If those words from the same topic cooccur very often (i.e., the 𝐶𝐶𝑉𝑉 coherence is high), the model performs well [24].

Previous studies have also indicated that this criterion has an appropriate performance in determining the number of topics and is closely related to human judgments about the interpretation of topics [25, 26].

The main words and titles, as well as abstracts of articles in each topic category, were extracted after implementing LDA techniques based on the number of topics obtained by the mentioned method (CV coherence measure). Afterward, the words of each topic category along with the title and abstract of the articles, were given to topic experts (three of the authors of this article are topic experts in this field). After reviewing the relevant data related to each category, these experts defined a label (name) for the topic categories.

Python programming language and text mining-related libraries such as Gensim, NLTK, and spaCy have been applied to implement these steps [27]. Python programming language is open source, small-size and versatile, has simple syntax, is straightforward to develop, and provides a variety of libraries for users to work with texts [27].

In the next step, after identifying the topics and the number of articles related to each topic, the keyword density map of each topic was obtained using the co-word analysis in VOSviewer software, which is one of the most important and frequently employed software for data analysis of citation databases. It clusters the most relevant documents and the relationships between them [28]. The VOSviewer provides an opportunity to draw maps based on Terms [29].

## 3. Results

**Figure 1:**
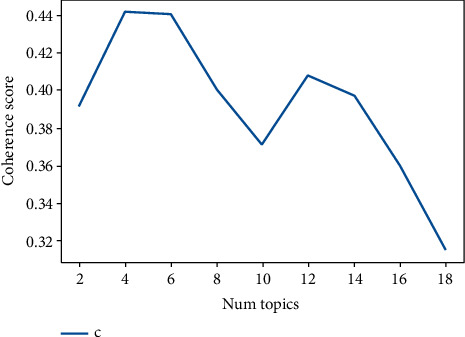
Value of CV coherence in the number of different topics.

A total of 159,490 articles in the field of tuberculosis were extracted from the Scopus database. The CV coherence was applied to select the number of topics in the LDA algorithm.  Figure 1 indicates the value of CV coherence in the number of topics.

Figure 1 shows the value of CV coherence between 2 and 40 topics, with the highest value of 4; therefore, four topics were selected for the topic modeling of publications on tuberculosis.

The results obtained from topic modeling in four main topics are indicated in Table 1, in which the topic's name, the number of articles on each topic, and the word cloud image are presented.

Table 1 indicates that the topic “Clinical symptoms,” with 41.8%, has the highest rate in the published articles on tuberculosis, followed by the topics “Diagnosis and treatment” with 28.1%, “Bacterial structure, pathogenicity, and genetics with 22.3%, and “Prevention” with 7.8%.

**Figure 2:**
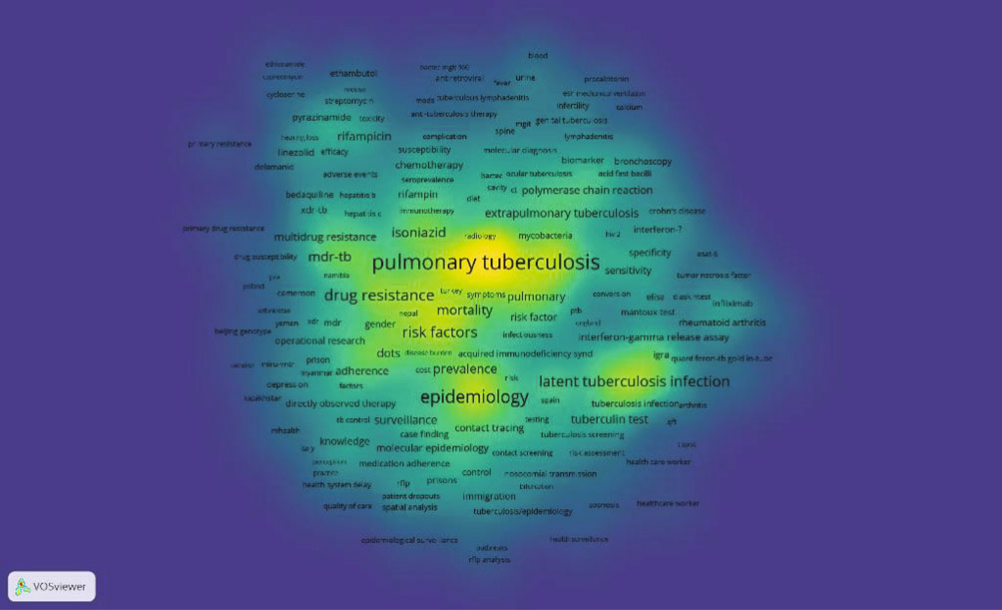
Densities of keywords in the topic “Diagnosis and treatment” in scientific articles on tuberculosis.

**Figure 3:**
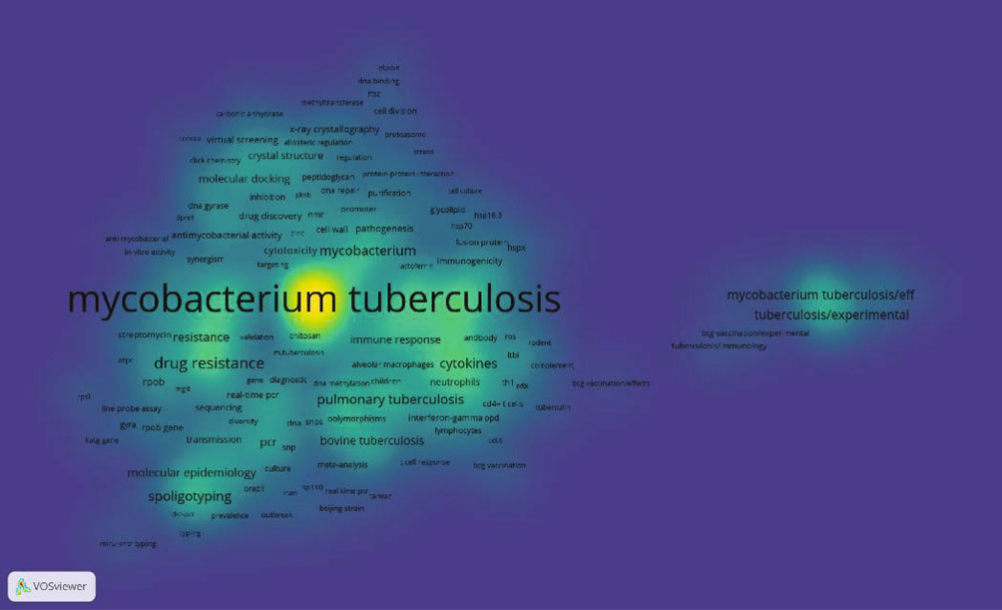
Densities of keywords in the topic “Clinical symptoms” in scientific articles on tuberculosis.

**Figure 4:**
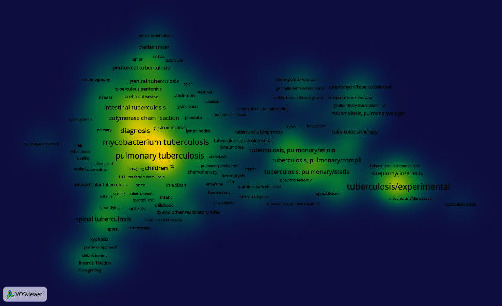
Densities of keywords in the topic “Diagnosis and treatment” in scientific articles on tuberculosis.

**Figure 5:**
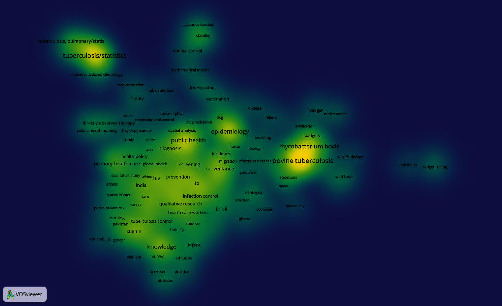
Densities of keywords in the topic “Prevention” in scientific articles on tuberculosis.

Figures 2–5 illustrate the densities of the author keywords used in the articles on each topic. In these figures, the range of colors from yellow to blue indicates the density of the keyword. The density weights are shown with yellow, green, and blue colors, respectively. There are frequently used keywords in the yellow sections, and in general, the higher the density, the map is seen with yellow color. Moreover, the closely related keywords have the most cooccurrence in published scientific articles on tuberculosis.

Figure 2 indicates that the keywords “Pulmonary tuberculosis,” “Epidemiology,” “drug-resistant,” “latent tuberculosis infection,” and “risk factors” have the highest densities in the topic “Diagnosis and treatment” in scientific articles on tuberculosis.

Figure 3 shows that the keywords “*Mycobacterium tuberculosis*,” “pulmonary tuberculosis,” “polymorphism,” “cytokines,” and “drug resistance” are among the high-density keywords in the topic “clinical symptoms” in scientific articles on tuberculosis.

Figure 4 demonstrates that in the topic “Diagnosis and treatment” in scientific articles on tuberculosis, the keywords “*Mycobacterium tuberculosis*,” “pulmonary tuberculosis,” and “tuberculosis/experimental” have the highest densities.

Figure 5 indicates that in the topic “Prevention” in scientific articles on tuberculosis, the keywords “bovine tuberculosis,” “*Mycobacterium bovis*,” “epidemiology,” “public health,” and “tuberculosis/statistics” have the highest densities.

**Figure 6:**
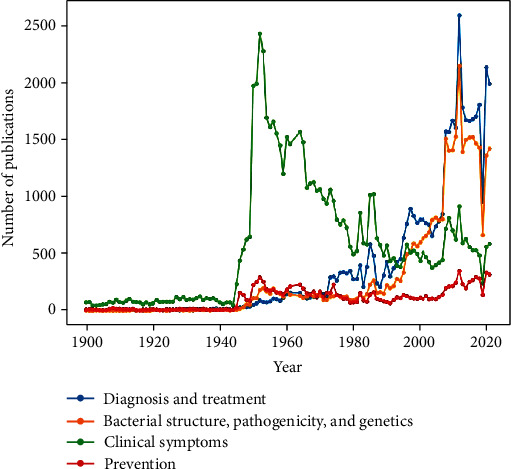
Publishing trend of scientific articles on tuberculosis from 1900 to 2021.

Moreover, Figure 6 demonstrates the publishing trend of four main topics of scientific articles on tuberculosis.

Figure 6 shows that the highest publication rates on the topics “Clinical symptoms,” “Diagnosis and treatment,” and “Bacterial structure, pathogenicity, and genetics” were in 1952, 2012, and 2012, respectively. Moreover, the topic “Prevention” has also had a constant publishing trend over time. According to Figure 6, the publication trend of each topic in the last 20 years indicates that in 2000, the most published topics were related to “Diagnosis and treatment” and “Bacterial structure, pathogenicity, and genetics,” respectively. Meanwhile, in 2003 and 2004, the number of publications on “Bacterial structure, pathogenicity, and genetics” was more than other topics. Moreover, in the last 20 years, the topics “Clinical symptoms” and “Prevention” have been placed after the other two topics.

## 4. Discussion

Tuberculosis is among the oldest and most important infectious diseases that, since its onset, has sent many people to the brink of death and incurred many medical and economic costs [30]. This disease is found all over the world and has suddenly increased in different periods in one or more geographical areas for different reasons, such as natural disasters, wars, the emergence of drug-resistant strains of bacteria, incomplete or lack of implementation of prevention programs (vaccination, DOTS plans, etc.), health and economic status of the community, weakened immune system due to stresses, consuming immune system weakening medications, poor nutrition, and the simultaneous occurrence of infectious and noninfectious diseases that suppress and weaken the immune system. Moreover, the WHO has formulated and implemented regional and global programs for the diagnosis, treatment, and prevention of this disease [31, 32].

In the present investigation, according to the results, the published articles on tuberculosis have been classified into four topics “Clinical symptoms,” “Diagnosis and treatment,” “Bacterial structure, pathogenicity, and genetics,” and “Prevention,” in respective order of publication trends of each topic from high to low, which covers all studies.

In this regard, Igwaran and Edoamodu identified three main clusters for tuberculosis publications in Africa between 2010 and 2019. This cluster included the topics (1) pulmonary tuberculosis as an indicator of the site of infection, tuberculosis as an indicator of the type of bacterial infection, (2) epidemiology as an indicator of the study, transmission as an indicator of disease spread, and (3) HIV as an indicator of concomitant infection [6]. Moreover, Nafade et al. indicated that tuberculosis publications between 2007 and 2016 are mainly in the subject areas of “Fundamental research” and “Epidemiology,” followed by “Operational and public health research,” “Diagnostics,” “Treatment,” and “Vaccines” [5]. Lee and Yim reported that research topics “diagnosis” (43.1%) and “treatment” (16.9%) had the highest number of publications, as well as “operational and public health research” and “vaccines” had the lowest number in South Korea [33]. Moreover, Dastani et al. indicated that the highest number of scientific publications in the field of brucellosis was prevention, clinical signs, and diagnosis topics [34].

The results of the present study reveal that the clinical symptoms of tuberculosis have been the most significant concern and challenge for scientists. Since 1900, the most research has been performed on the clinical symptoms of tuberculosis, which can be due to the unknown nature of different dimensions of clinical symptoms of the disease at that time as well as the difficulty in distinguishing it from infectious or noninfectious diseases with similar symptoms [35, 36]. Furthermore, the high number of articles on clinical symptoms can be due to the concern of physicians and specialists in the correct diagnosis of tuberculosis. On the other hand, some clinical symptoms of this disease are similar to those of some other infectious diseases. Therefore, it is essential to distinguish tuberculosis from other diseases. In this case, the physician can prescribe the correct and timely diet for the patient. Consequently, treatment costs and other complications due to misdiagnosis will not be imposed on the patient. Furthermore, the most publications on the topic of clinical symptoms were in 1952, and after that, the rate of publications on this topic decreased.

Due to the relatively high prevalence and mortality caused by tuberculosis during the years before 1952, countries and the WHO have focused on the correct diagnosis of the disease and its clinical symptoms; therefore, since 1952, the death rate due to tuberculosis has been declining more steeply. In 1952, the number of people who visited hospitals and medical centers also increased, and on the other hand, physicians focused more on identifying and controlling tuberculosis patients. Moreover, the tuberculosis vaccination program was implemented in some countries where this disease was common. The discovery of isoniazid in 1952 as the first and most effective oral antimycobacterial drug is another reason for focusing on research and correct diagnosis of patients and their treatment [37].

The results of the present study demonstrated that the topic “diagnosis and treatment” has dedicated the second most published articles since 1900; and in 2012, the highest number of research papers were published in this field. Since the diagnosis and treatment of tuberculosis has been among the main challenges of the health system for tuberculosis patients from the beginning, these results can be justified. Moreover, due to incorrect diagnosis and clinical similarities with some other diseases, as well as nonuse or misuse of antituberculosis drugs, drug resistance in this bacterium has occurred over time, this issue was one of the main challenges in the world in 2012, and many studies have been performed in this field [38]. Currently, the WHO is implementing detailed plans to cope with the problem of drug resistance in this bacterium at a global level.

The results of the present study indicated that the study on the structure of bacteria in 2012 has been at the highest level, which could be in line with the world's efforts to better understand the structure of bacteria, mechanisms of bacterial resistance, pathogenicity, the use of new and up-to-date techniques to fully understand how the immune system responds to the structural components of bacteria, designing new diagnostic tests, and producing vaccines.

## 5. Conclusion

The results of the present investigation revealed that since 1900, researchers have consistently and continuously conducted numerous studies on the prevention of tuberculosis, and its chart follows a fixed pattern, which shows that since then, human beings have constantly been looking for effective and efficient ways to prevent this disease and offer new prevention programs. Although the advancement of human beings and the possibility of more effortless movement and communication has made it difficult to control the disease, fortunately, with the progress of science and studies, more up-to-date and advanced programs and protocols have been proposed to prevent this disease.

Considering that scientific publications are increasing daily, it is impossible for experts to read all of the literature of a scientific field; therefore, it is important to use automatic knowledge extraction techniques from big data to improve the knowledge of experts.

According to the study results, the four research topics were extracted from the tuberculosis research. An understanding of current research trends and topics helps to find the gaps that should be further studied.

The findings of the present research can be useful for experts, physicians, and decision-makers to plan to control tuberculosis and also help physicians in making proper decisions for patients. Healthcare organizations can also use the data of this study in precise planning in regional health centers to fight tuberculosis. The WHO is constantly collecting up-to-date data on various diseases, including tuberculosis. The results of the present investigation can be helpful for this organization to make important decisions, be informed of the process of studies on tuberculosis, as well as invest in and formulate appropriate plans and guidelines against this disease.

## Figures and Tables

**Table 1:** Topics obtained from topic modeling of scientific articles on tuberculosis.

Name (rate/percentage)	Word cloud
Diagnosis and treatment (44788/28.1%)	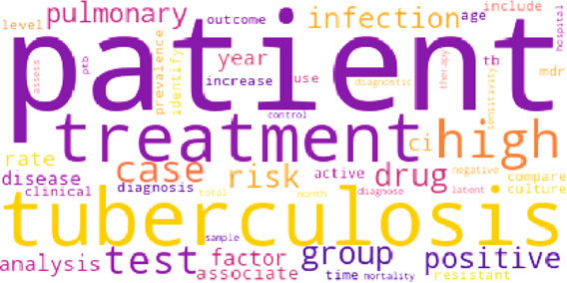
Bacterial structure, pathogenicity, and genetics (35524/22.3%)	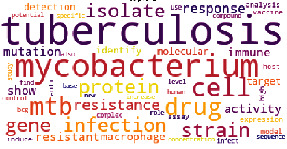
Clinical symptoms (66667/41.8%)	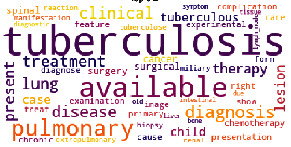
Prevention (12511/7.8%)	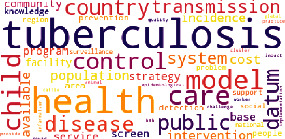

## Data Availability

The data that support the findings of this study are available from the corresponding author upon reasonable request.
